# Inhibitory Effects Induced by *Vicia faba*, *Uncaria rhyncophylla*, and *Glycyrrhiza glabra* Water Extracts on Oxidative Stress Biomarkers and Dopamine Turnover in HypoE22 Cells and Isolated Rat Striatum Challenged with 6-Hydroxydopamine

**DOI:** 10.3390/antiox8120602

**Published:** 2019-11-29

**Authors:** Giustino Orlando, Annalisa Chiavaroli, Sheila Leone, Luigi Brunetti, Matteo Politi, Luigi Menghini, Lucia Recinella, Claudio Ferrante

**Affiliations:** Department of Pharmacy, Università degli Studi “Gabriele d’Annunzio”, via dei Vestini 31, 66100 Chieti, Italy; giustino.orlando@unich.it (G.O.); annalisa.chiavaroli@unich.it (A.C.); sheila.leone@unich.it (S.L.); luigi.brunetti@unich.it (L.B.); matteo.politi@unich.it (M.P.); lucia.recinella@unich.it (L.R.); claudio.ferrante@unich.it (C.F.);

**Keywords:** Parkinson’s disease, *Vicia faba*, *Glycyrrhiza glabra*, *Uncaria rhyncophylla*, l-dopa, dopamine

## Abstract

Background: Parkinson’s disease (PD) is the most common and progressive neurodegenerative and oxidative stress-related disorder, characterized by a dramatic loss of dopamine (DA) neurons in the nigrostriatal tissue. The first-line drug for PD treatment is represented by l-dopa, although clinical and preclinical studies pointed out the potential efficacy of medicinal plant- and food-derived antioxidants as brain protective agents. In this regard, the potential application of *Vicia faba*, *Uncaria rhyncophylla*, and *Glycyrrhiza glabra* extracts is of noteworthy interest, despite a lack of information in the scientific literature as regards their effect on striatal DA level. Methods: The protective effects of *V. faba, U. rhyncophylla*, and *G. glabra* water extracts were investigated on HypoE22 cells and isolated rat striatum specimens challenged with 6-hydroxydopamine (6-OH-DA). The extract effects against lactate dehydrogenase (LDH), nitrites, and 8-iso-prostaglandin(PG)F2α were evaluated using either single-extract treatments or a treatment with a pharmacological association. Additionally, the turnover of DA was measured. Results: The pharmacological association of the extracts was the most effective in contrasting the upregulated LDH and nitrite levels and in reducing striatal DA turnover. Conclusion: The present findings corroborate the rational for the traditional use of *V. faba*, *G. glabra*, and *U. rhyncophylla* extracts, supporting their pharmacological association in order to improve their protective effects.

## 1. Introduction

Oxidative/nitrosative stress plays a master role in age-related cell degeneration, particularly in the brain, where the increased burden of peroxide-modified unsaturated fatty acids is coupled with the inhibition of antioxidant systems [1,2]. The adult brain displays a high level of unsaturated fatty acids which are highly sensitive to peroxidation induced by mitochondrial-derived reactive oxygen/nitrogen species (ROS/RNS) [3]. The intrinsic modest antioxidant defense coupled with neurotransmitter autoxidation is also causally related to the brain susceptibility to oxidative/nitrosative stress [4]. Parkinson’s disease (PD) is the most common and progressive neurodegenerative and oxidative stress-related disorder, characterized by a dramatic loss of dopamine (DA) neurons in the nigrostriatal tissue, which in turn causes impairment in voluntary movement control [5]. Actually, more than 10 million people worldwide are affected by PD, with the incidence increasing progressively with age, despite there being 4% of PD patients under 50 years of age, particularly in the male population [6]. The first-line drug is represented by l-dopa that is able to contrast, albeit partially, the loss of DA in the nigrostriatal tissue [7]. Nevertheless, long-term benefits related to l-dopa administration are inconsistent [8]. For this purpose, l-dopa is co-administered with amino acid decarboxylase inhibitors, including carbidopa and benserazide, in order to reduce the peripheral conversion of l-dopa to DA and the consequent peripheral (nausea, emesis) and central (dyskinesia, on–off phenomenon) side effects [5]. In PD, the activity of monoamino oxidase B (MAO-B) is also increased, thus leading to both DA depletion and increased mitochondrial-derived ROS production [7]. Clinical and preclinical studies pointed out the potential efficacy of medicinal plant- and food-derived antioxidants as protective agents in the brain of both young and aged rats [9,10]. Additionally, herbal extracts and isolated phytocompounds able to counteract the burden of oxidative stress and inflammation in the brain revealed efficacy in restoring tissue neurotransmitter levels in both in vitro and in vivo experimental models of neurodegeneration, possibly through multiple concomitant mechanisms [10,11,12]. Furthermore, considering the multifactorial metabolic aspects of PD, the pharmacological association of medicinal plant-derived extracts could ameliorate their efficacy [13]. Several herbal extracts were found to be effective in experimental models of PD, particularly for their capability to contrast the increased burden of inflammation/oxidative stress and improve DA signaling [6]. In this regard, the potential application of *Vicia faba*, *Uncaria rhyncophylla*, and *Glycyrrhiza glabra* extracts as adjuvant agents in the management of clinical symptoms related to PD is of noteworthy interest. *V. faba* represents the elective natural source of l-dopa, which was isolated from beans by Torquato Torquati in 1910–1911, and whose structure was described by Markus Guggenheim in 1913. Case report studies also indicated the efficacy of *V. faba* beans in improving the motor function in PD patients, through the prolonging of “on” periods, following the ingestion of a large bean meal [14]. A recent double-blind clinical trial also pointed out the efficacy of a polyphenol-rich *G. glabra* extract, administered as adjuvant therapy, in improving the motor function in PD patients [15]. *U. rhyncophylla*, besides being traditionally used in Northern Asia as an anti-Parkinson remedy, was shown to be effective as a neuroprotective agent in PC12 cells challenged with 6-hydroxydopamine (6-OH-DA), a well-recognized pro-inflammatory and pro-degenerative stimulus employed in in vitro and in vivo models of PD [16]. Nevertheless, there is still a lack in the scientific literature as regards the putative role of these medicinal plants in modulating DA levels in the striatum. With the aim to further elucidate the mechanism of action of these herbs in PD treatment, in the present work, the protective effects of *V. faba, U. rhyncophylla*, and *G. glabra* water extracts were investigated in an experimental model of neurotoxicity consisting of neuronal HypoE22 cells and isolated rat striatum specimens challenged with 6-OH-DA. The protective effects of the extracts were evaluated by analyzing selected biomarkers of cytotoxicity and nitrosative and oxidative stress, namely, lactate dehydrogenase (LDH), nitrites, and 8-iso-prostaglandin(PG)F_2α_, respectively, using both single-extract treatments and a pharmacological association (PARKININAX^®^). In the same condition, DA turnover was measured as well and expressed as the ratio between dihydroxyphenilacetic acid (DOPAC) and DA levels. Finally, in order to provide a better interpretation of the observed pharmacological effects, a fingerprint analysis was carried out on selected phenolic compounds, namely, gallic acid, catechin, epicatechin, and resveratrol, which are known to exert protective effects at both central and peripheral level via multiple mechanisms. In this regard, gallic acid, besides having anti-radical effects, especially at low to moderate concentrations [17,18], was recently described to exert protective effects in an experimental PD model in vitro [19]; in addition, catechin intake was related to a lower risk of PD, possibly through a regulatory effect on neuronal viability and synaptic plasticity [20]. Finally, resveratrol displayed an intriguing efficacy against PD both in vitro [21] and in vivo [22], being also able to synergize with l-dopa [23].

## 2. Materials and Methods 

### 2.1. Plant Material

Commercial water extracts were obtained from the roots of *G. glabra* L. (standardized in glycirrhizic acid 21% *w/w),* the hooks of *U. rhynchophylla* (Miq.) Miq. ex Havil., the beans of *V. faba* L., and the registered trademark formula PARKININAX^®^ (*V. faba*/*G. glabra*/*U. rhyncophylla* 85:10:5), kindly provided as dried materials by Cristalfarma S.r.l. (Milan, Italy). Just before the phytochemical and pharmacological assays, the extracts were rehydrated in bidistilled water through a Trans-sonic T460 ultrasonic bath supplied by Elma (Singen, Germany) for 10 min at room temperature and full power (35 kHz), as previously described [24].

### 2.2. Phytochemical Analysis

*G. glabra*, *U. rhyncophylla*, and *V. faba* extracts (5 µg/mL) were analyzed for phenol quantitative determination using a reversed-phase HPLC–fluorimeter in gradient elution mode. The analyses were carried out by using a liquid chromatograph (MOD. 1525, Waters Corporation, Milford, MA, USA) equipped with a fluorimetric detector (MOD. 2475, Waters Corporation), a C18 reversed-phase column (AcclaimTM 120, 3 µm, 2.1 × 100 mm, Dionex Corporation, Sunnyvale, CA, USA), and an on-line degasser (Biotech 4-CH degasi compact, LabService, Anzola Emilia, Italy). The gradient elution was achieved by a mobile phase consisting of methanol/acetic acid/water (10:2:88, *v/v*) as solvent A and methanol/acetic acid/water (10:2:88, *v/v*) as solvent B, in agreement with an already published paper [25]. According to the same authors, λ_ex_ = 278 nm and λ_em_ = 360 nm wavelengths were selected in order to analyze the following phenolic compounds: gallic acid, catechin, epicatechin, and resveratrol.

### 2.3. Artemia salina Lethality Test

The *Artemia salina* lethality bioassay was performed as previously reported [26]. Briefly, brine shrimp larvae were bred at 25–28 °C for 24 h in the presence of *G. glabra*, *U. rhyncophylla*, and *V. faba* extracts (0.1–20 mg/mL) dissolved in incubation medium (artificial sea water). After an incubation period of 24 h with the extracts, the number of surviving shrimps was evaluated, and the mortality percentage was calculated with the following equation: ((T − S)/T) × 100%, where T and S are the total number of incubated larvae and surviving nauplii, respectively. The xperiments were carried out in triplicate. 

### 2.4. Cell Cultures and Viability Test

Hypo-E22 cells were purchased from Cedarlane Cellution Biosystem (Burlington, ON, Canada) and cultured in DMEM supplemented with 10% (*v/v*) heat-inactivated fetal bovine serum and 1.2% (*v/v*) penicillin G/streptomycin in 75 cm^2^ tissue culture flask (*n* = 5 individual culture flasks for each condition). The experimental conditions were fully described in our previous paper [27]. To assess the basal cytotoxicity of the extracts, a viability test was performed in 96-microwell plates, using the 3-(4,5-dimethylthiazol-2-yl)-2,5-diphenyltetrazolium bromide (MTT) test. Cells (2500 per microwell) were incubated with the extracts (ranging concentration 5–100 μg/mL) for 24 h. After the treatment period, 10 μL of MTT (5 mg/mL) was added to each well, and the cells were incubated for 3 h. The formazan dye formed was extracted with dimethyl sulfoxide, and the absorbance was recorded as previously described [28]. 

### 2.5. Ex Vivo Pharmacological Study

Twenty-four Sprague-Dawley rats (200–250 g) were housed in Plexiglass cages (40 cm × 25 cm × 15 cm), two rats per cage, in climatized colony rooms (22 ± 1 °C; 60% humidity), on a 12 h/12 h light/dark cycle (light phase: 7:00–19:00), with free access to tap water and food, 24 h/day throughout the study, with no fasting periods. The rats were fed a standard laboratory diet (3.5% fat, 63% carbohydrate, 14% protein, 19.5% other components without caloric value; 3.20 kcal/g). Housing conditions and experimentation procedures were strictly in accordance with the European Union ethical regulations on the care of animals for scientific research. The experiments were approved by the Local Ethical Committee (University “G. d’Annunzio” of Chieti-Pescara) and the Italian Health Ministry (Italian Health Ministry authorization N. F4738.N.XTQ, delivered for the period 11 November 2018–11 November 2019). The rats were sacrificed by CO_2_ inhalation (100% CO_2_ at a flow rate of 20% of the chamber volume per min), and striatum specimens were immediately collected and maintained at 37 °C for 4 h in RPMI supplemented with 6-OH-DA (100 µM). During the incubation period, the striatum specimens were separately treated with the extracts of *U. rhyncophylla*, *G. glabra*, *V. faba* (5, 10, 85 µg/mL, respectively), or PARKININAX^®^ (100 µg/mL). Afterwards, the striatum tissues were homogenized in a 50 mM perchloric acid solution for biochemical determinations, as described below.

### 2.6. Striatum Levels of Nitrites

Nitrites are stable nitric oxide end products, whose determination is commonly used as an index of nitric oxide production in vivo. Briefly, nitrite production was determined by mixing 50 µL of the assay buffer with 50 µL of Griess reagent (1.5% sulfanilamide in 1 M HCl plus 0.15% *N*-(1-naphthyl) ethylenediamine dihydrochloride in distilled water, *v/v*). After 10 min incubation at room temperature, the absorbance at 540 nm was determined, and nitrite concentrations were calculated from a sodium nitrite standard curve. All measurements were performed in triplicate.

### 2.7. 8-iso-PGF_2α_ Radioimmunoassay

Specific anti-8-iso-PGF2α was developed in rabbit; its cross-reactivity with 8-iso-PGE2 is 7.7%, whereas that with other prostanoids is <0.3% [29]. In this experiment, 100 μL of prostaglandin standard or sample were incubated overnight at 4 °C with ^3^H-prostaglandin (3000 cpm/tube; NEN) and the antibody (final dilution 1:120,000; kindly provided by Prof. G. Ciabattoni), in 1.5 mL of 0.025 M phosphate buffer. Free and antibody-bound prostaglandins were separated by the addition of 100 μL of 5% bovine serum albumin and 100 μL of 3% charcoal suspension, followed by centrifuging for 10 min at 4000× *g* at 5 °C and decanting the supernatants into the scintillation fluid (Ultima Gold™, Perkin Elmer Italia, Milan, Italy) for β-emission counting. The detection limit of the assay method was 0.6 pg/mL. The IC_50_ was 39.8 pg/mL. The intra-assay and inter-assay coefficients of variation were ±2.0% and ±2.9% at the lowest concentration of standard (2 pg/mL) and ±3.7% and ±9.8% at the highest concentration of standard (250 pg/mL).

### 2.8. Lactate Dehydrogenase Assay

LDH activity was measured by evaluating the consumption of NADH in 20 mM HEPES-K^+^ (pH 7.2), 0.05% bovine serum albumin, 20 μM NADH and 2 mM pyruvate, using a microplate reader (excitation wavelength 340 nm, emission wavelength 460 nm) according to the manufacturer’s protocol (Sigma-Aldrich Italia, Milan, Italy). Data were from triplicate tests and were expressed as relative variations compared to the vehicle-treated group.

### 2.9. Neurotransmitter Extraction and HPLC–EC Determination

Striatum DA and DOPAC and l-dopa content in *V. faba* were determined by extraction in 50 mM perchloric acid solution, as previously reported [30]. Afterwards, their levels were analyzed through an HPLC apparatus consisting of a Jasco (Tokyo, Japan) PU-2080 chromatographic pump and an ESA (Chelmsford, MA, USA) Coulochem III coulometric detector, equipped with a microdialysis cell (ESA-5014b) porous graphite working electrode and a solid-state palladium reference electrode. The analytical conditions for biogenic amine identification and quantification were selected as previously reported [30]. Briefly, the analytical cell was set at −0.150 V for detector 1 and at +0.300 V for detector 2, with a range of 100 nA. The chromatograms were monitored at the analytical detector 2. Integration was performed by the Jasco Borwin Chromatography software, version 1.5. Chromatographic separation was performed by isocratic elution on a Phenomenex Kinetex reverse-phase column (C18, 150 mm × 4.6 mm i.d., 2.6 µm). The mobile phase was (10:90, *v/v*) acetonitrile and 75 mM phosphate buffer pH 3.00 containing octanesulfonic acid 1.8 mM, EDTA 30 µM, and triethylamine 0.015% (*v/v*). The flow rate was 0.6 mL/min, and the samples were manually injected through a 20 µL loop. Neurotransmitter peaks were identified by comparison of their retention times with those of pure standards. Neurotransmitter concentrations in the samples were calculated by a linear regression curve (*y* = b*x* + m) obtained using the standards. Neither internal nor external standards were necessary for neurotransmitter quantification in the striatum homogenate, and all tests performed for method validation yielded results in accordance to the limits indicated in official guidelines for applicability in laboratory trials. The standard stock solutions of DA, l-dopa, and DOPAC at 2 mg/mL were prepared in bidistilled water containing 0.004% EDTA and 0.010% sodium bisulfite. The stock solutions were stored at 4 °C. Work solutions (1.25–20.00 ng/mL) were obtained daily, by progressively diluting the stock solutions in the mobile phase. 

### 2.10. Statistical Analysis

Statistical analysis was carried out through GraphPad Prism version 5.01 for Windows (GraphPad Software, San Diego, CA, USA). Means ± S.D. were determined for each experimental group and analyzed by one-way analysis of variance (ANOVA), followed by Newman–Keuls comparison multiple test. Statistical significance was set at *p* < 0.05. As regards the animals employed for the experiments, their number per condition (*n* = 4) was calculated with the software G*Power (v3.1.9.4, University of Kiel, Kiel, Germany). The values of study potency (1−β) and significance level (α) were 0.8 and 0.05, respectively.

## 3. Results

### 3.1. Phytochemical Analysis

The HPLC–fluorimetric analysis was focused on selected phenolic compounds, namely, gallic acid, catechin, epicatechin, and resveratrol. The results indicated that the *U. rhyncophylla* water extract was the richest in gallic acid, whereas the *G. glabra* water extract displayed the best qualitative profile alongside with higher levels of catechin, epicatechin, and resveratrol (Table 1). Conversely, the *V. faba* water extract displayed the poorest qualitative and quantitative profiles, as regards the selected phenolic compounds. The *V. faba* extract was also assayed via HPLC–EC for quantifying its level of l-dopa (33.71 ± 4.21 µg/g dry extract). The results of the HPLC-fluorimetric analysis were consistent with the colorimetric evaluations of total phenols (expressed as mg of gallic acid per g of dry extract) previously described in the literature [31,32,33], although a punctual description of the *U. rhyncophylla* phenolic profile is still lacking. The concentration of l-dopa found in the *V. faba* extract was consistent with the content of this compound in green plant pods [34].

### 3.2. Pharmacological Studies

**Figure 1:**
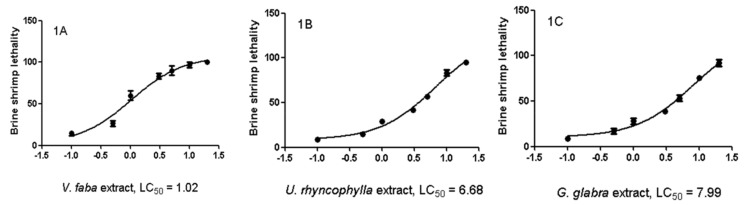
Brine shrimp lethality assay on *V. faba* (**A**), *U. rhyncophylla* (**B**), and *G. glabra* (**C**). the LC_50_ values were 1.02, 6.68, and 7.99 mg/mL, respectively.

**Figure 2:**
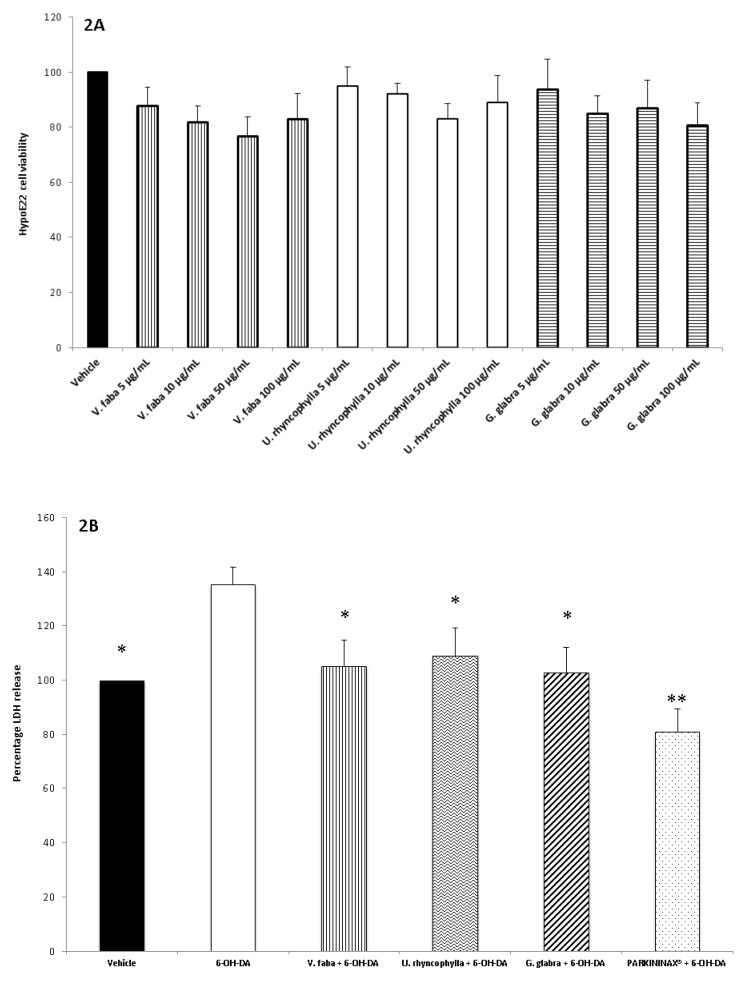
(**A**) Effect of *G. glabra*, *U. rhyncophylla*, and *V. faba* water extracts (5–100 µg/mL) on Hypo-E22 cells viability (MTT test). (**B**) Effect of *G. glabra* (10 µg/mL), *U. rhyncophylla* (5 µg/mL), and *V. faba* (85 µg/mL) water extracts and PARKININAX^®^ (100 µg/mL) on 6-hydroxydopamine (6-OH-DA)-induced lactate dehydrogenase (LDH) level (cytotoxicity test) in Hypo-E22 cells. Data are reported as percentage of LDH release (mU/mL) with respect to Vehicle, defined as 100%. (ANOVA, *p* < 0.001; post-hoc, ** *p* < 0.01, * *p* < 0.05 vs. 6-OH-DA).

**Figure 3:**
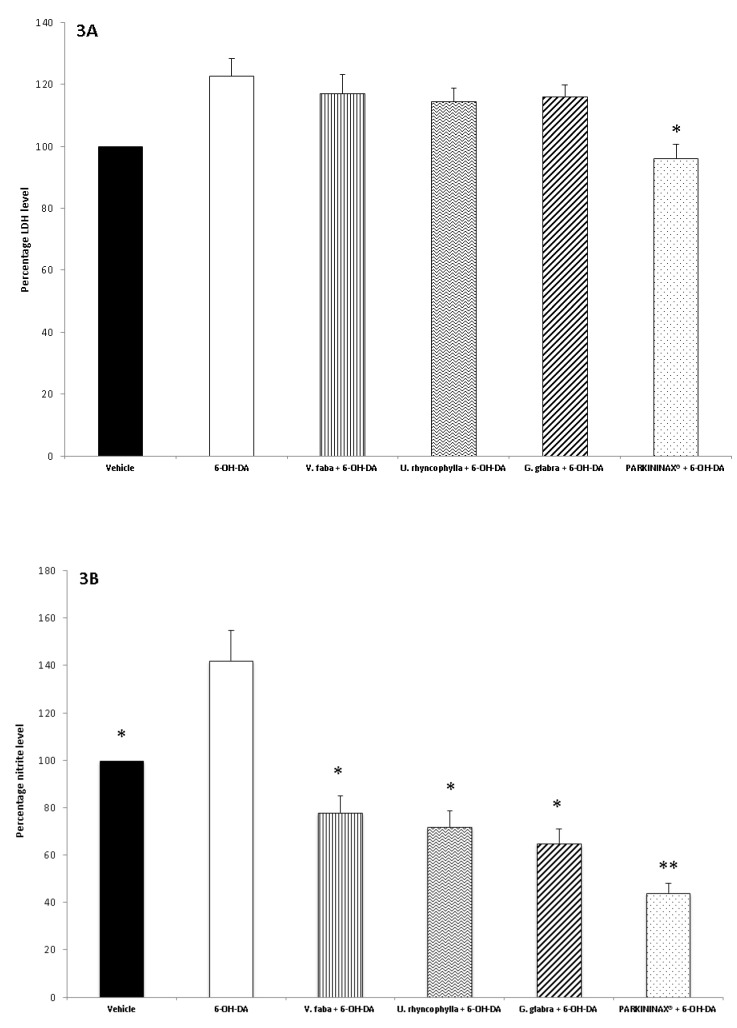

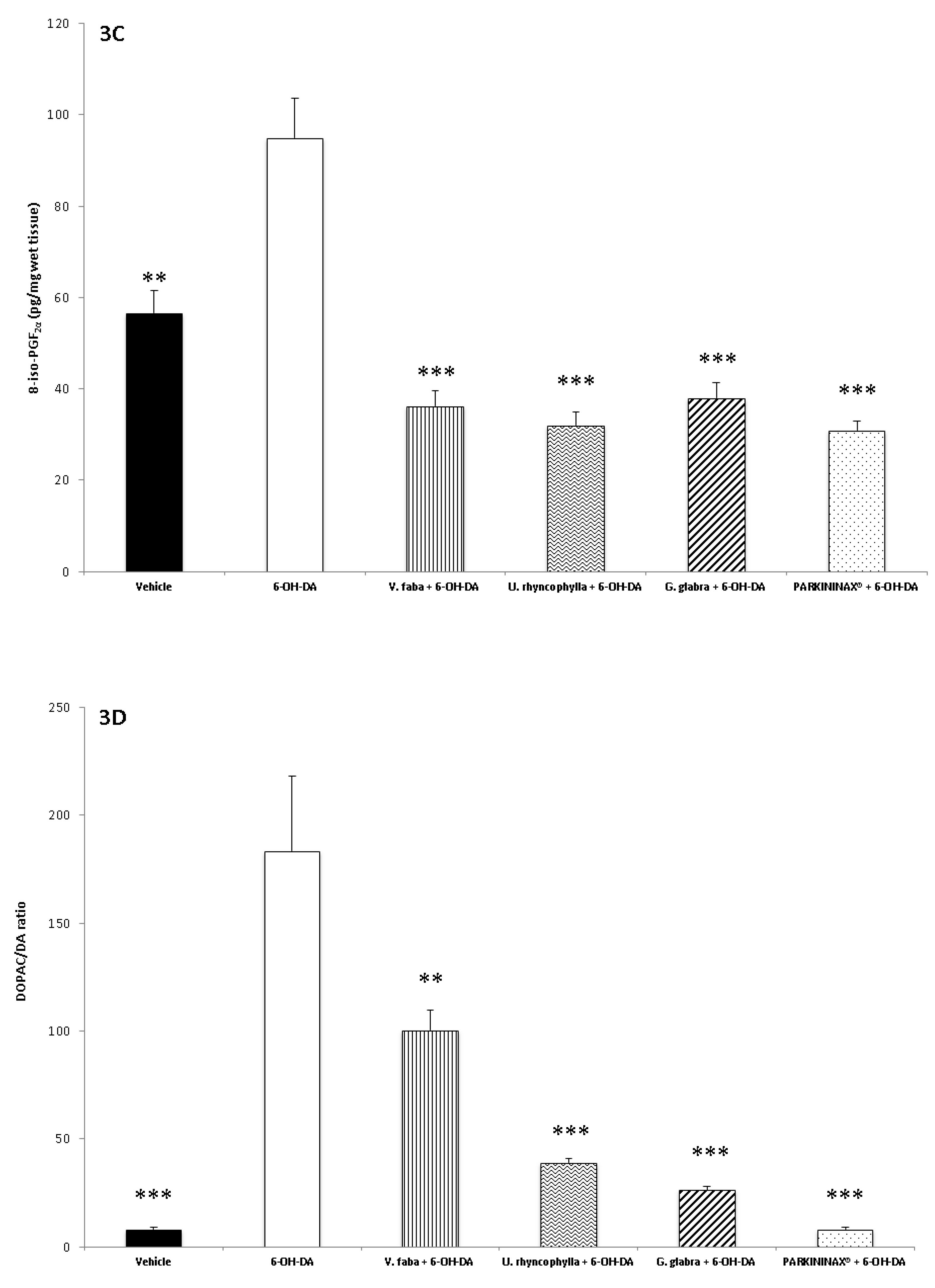
(**A**) Effect of *G. glabra* (10 µg/mL), *U. rhyncophylla* (5 µg/mL), and *V. faba* (85 µg/mL) water extracts and PARKININAX^®^ (100 µg/mL) on 6-OH-DA-induced LDH in isolated rat striatum specimens. Data are reported as percentage of LDH (mU/mg wet tissue) with respect to the Vehicle group. (ANOVA, *p* < 0.05; post-hoc, * *p* < 0.05, vs. 6-OH-DA). (**B**) Effect of *G. glabra* (10 µg/mL), *U. rhyncophylla* (5 µg/mL), and *V. faba* (85 µg/mL) water extracts and PARKININAX^®^ (100 µg/mL) on 6-OH-DA-induced nitrite levels in isolated rat striatum specimens. Data are reported as percentage of nitrite (µg/mg wet tissue) with respect to Vehicle, defined as 100%. (ANOVA, *p* < 0.001; post-hoc, ** *p* < 0.01, * *p* < 0.05 vs. 6-OH-DA). (**C**) Effect of *G. glabra* (10 µg/mL), *U. rhyncophylla* (5 µg/mL), and *V. faba* (85 µg/mL) water extracts and PARKININAX^®^ (100 µg/mL) on 6-OH-DA-induced 8-iso-prostaglandin(PG)F_2α_ level (pg/mg wet tissue) in isolated rat striatum specimens (ANOVA, *p* < 0.0001; post-hoc, ** *p* < 0.01, *** *p* < 0.001 vs. 6-OH-DA). (**D**) Effect of *G. glabra* (10 µg/mL), *U. rhyncophylla* (5 µg/mL), and *V. faba* (85 µg/mL) water extracts and PARKININAX^®^ (100 µg/mL) on 6-OH-DA-induced dopamine turnover (dihydroxyphenilacetic acid/dopamine (DOPAC/DA) ratio) in isolated rat striatum specimens. DA and DOPAC levels are expressed as ng/mg wet tissue. (ANOVA, *p* < 0.0001; post-hoc, ** *p* < 0.01, *** *p* < 0.001 vs. 6-OH-DA).

The three extracts were tested for their neuroprotective role after evaluating their biocompatibility (0.1–20 mg/mL) through a lethality test in the brine shrimp *A. salina* Leach. Since the results indicated LC_50_ values >1 mg/mL (Figure 1), a concentration range at least 10-fold lower was selected for the in vitro viability (MTT) assay on the HypoE22 cell line. Particularly, the MTT test revealed that all the three extracts (5–100 µg/mL) either alone or in pharmacological association (Formula) did not significantly modify HypoE22 cell viability, with a percentage viability in the range of 70–100% (Figure 2A). Considering the results of the MTT test and the aforementioned ratio of the single ingredients in the formula (PARKININAX^®^), the following extract concentrations were selected for the subsequent in vitro and ex vivo assays in HypoE22 cells and isolated rat striatum specimens challenged with 6-OH-DA: *V. faba*, 85 µg/mL; *G. glabra*, 10 µg/mL; *U. rhyncophylla*, 50 µg/mL. Being PARKININAX^®^ a classical pharmacological association, the resulting concentration was the algebric sum (100 µg/mL) of the concentrations of the selected extracts used in single treatments. Within the employed in vitro and ex vivo experimental paradigms, selected biomarkers of citotoxicity and nitrosative and oxidative stress, namely, LDH, nitrites, and 8-iso-PGF_2α_, respectively, were measured. The results indicated that the whole Formula was the most effective in contrasting the upregulated LDH and nitrite levels in HypoE22 cells and isolated rat striatum specimens, respectively (Figure 2B and Figure 3A,B revised), whereas the single extracts and the formula displayed comparable blunting effects on the 6-OH-DA-induced level of 8-iso-PGF_2α_ (Figure 3C). In the same condition, we also measured the turnover of DA, expressed as the DOPAC/DA ratio. The results depicted in Figure 3D indicate that the *V. faba* extract was the least effective in preventing the 6-OH-DA-induced turnover of DA, whereas *G. glabra* and *U. ryncophylla* displayed comparable efficacy. On the other hand, the formula was the most effective in inhibiting DA turnover in rat striatum.

## 4. Discussion

PD is a multifactorial disorder deeply related to an increased burden of oxidative/nitrosative stress, especially during aging [5]. PD is characterized by cellular degeneration in the striatum, where the increased level of peroxidized unsaturated fatty acids is paralleled by the progressive deficit of endogenous antioxidant systems [1,2]. The elevated levels of brain unsaturated fatty acids could be peroxidized by mitochondrial metabolism-derived ROS/RNS [3], and lipid peroxidation is well known to be linked to neurodegeneration [35,36,37]. On the other hand, preclinical studies have long suggested the importance of natural antioxidants as preventive agents of brain lipid peroxidation, particularly in pro-oxidant conditions [10,29]. Aging and neurodegenerative disorders are also characterized by a significant decrease in the levels of monoaminergic neurotransmitters, which in turn could explain, albeit partially, the progressive impairment in motor and cognitive functions [10,11,12]. In the present study, a multidirectional approach was followed in order to characterize the water extracts of selected traditional medicinal plants commonly used as antioxidants. Consistently with their quali–quantitative phenolic profile (Table 1), they have been long considered to be effective as protective agents in different PD experimental settings [14,15,16]. Through the lethality test in the brine shrimp and the MTT viability assay in the HypoE22 cell line, we selected the extract concentration (5–85 µg/mL) for the pharmacological tests. This biocompatibility limit, especially if referred to the *G. glabra* extract, is consistent with the findings of Azizsoltani and colleagues [38]. In the HypoE22 cell line, we also investigated the protective effects of the extracts and the formula against 6-OH-DA-induced cytotoxicity. We observed that the extracts and the formula were able to protect the cell line against 6-OH-DA-induced LDH, a significant marker of cytotoxicity and tissue damage [39]. The efficacy of the extracts was consistent with that reported in the literature [40]. Consistently with its higher content in secondary metabolites, the formula was obviously more effective than the single ingredients in inhibiting LDH increase [26,41]. In analogy, we observed a different pattern of antioxidant effects exerted by the formula and the single extracts in isolated striatum specimens challenged with 6-OH-DA. Specifically, the formula displayed the highest efficacy in inhibiting the induction of LDH, 8-iso-PGF_2α_, and nitrites by 6-OH-DA. The molecule 8-iso-PGF_2α_, derived from ROS/RNS peroxidation of membrane arachidonic acid, has long been considered a stable marker of lipid peroxidation and tissue damage in vivo [35], while herbal extracts rich in phenolic compounds with radical scavenger properties were revealed to be effective in blunting isoprostane production in ex vivo models of neurodegeneration [29,42,43]. On the other hand, nitrites represent a stable marker of nitrosative stress, which is strictly related to lipid peroxidation [44]. In PD, their levels reflect the upregulation of inducible nitric oxide synthase (iNOS), which is known to be inhibited by the catechin fraction of green tea and by resveratrol analogs [6,45]. Nevertheless, according to the cited literature, the effective iNOS inhibitory concentrations of these secondary metabolites appear to be higher compared to those measured in the single studied extracts, thus suggesting that the observed efficacy could involve other unidentified metabolites as well. In the isolated striatum, we also investigated the effects of the single extracts and the whole formula on DA turnover, expressed as DOPAC/DA ratio. The same ratio has long been considered an index of the activity of MAO-B, as well, whose upregulation is increased during aging and neurodegenerative diseases [46]. We observed that all tested extracts and the formula inhibited, albeit in part, 6-OH-DA-induced DA turnover. Specifically, the formula, consistently with its richest phenolic content aforementioned, was able to totally blunt 6-OH-DA-induced DA turnover in the rat striatum, while the single extracts showed partial capability in preventing the increase in the DOPAC/DA ratio, with *G. glabra* extract being the most promising. This is consistent with *G. glabra*’s higher content in epicatechin and catechin, that were reported to act as MAO-B inhibitors [47]. Conversely, the *V. faba* extract was the least effective in limiting the DOPAC/DA ratio. This result, despite appearing paradoxical in consideration of the l-dopa content in the *V. faba* phytocomplex, could be explained by both its lowest content in catechin and epicatechin and the intrinsic pro-oxidant effect of l-dopa itself. As a DA precursor, l-dopa, once administered, is able to promote not only the synthesis of DA but also its auto-oxidation, thus further supporting the association of antioxidants with this drug for long-term therapy of PD [5]. Additionally, despite there being clinical evidence indicating a significant increase in plasma DA concentrations following *V. faba* derivative administration [48], these studies did not include a functional dopaminergic evaluation in order to confirm the putative increased DA steady-state concentration in the striatum. Finally, considering the extensive l-dopa metabolism in peripheral tissues [5], the hypothetical increase of striatal DA following the sole administration of *V. faba*, without the co-administration of selective amino acid decarboxylase inhibitors, is questionable. On the other hand, we cannot exclude that the improvement in motor functions described in case report studies [14] could be the result of multiple antioxidant effects following *V. faba* treatment, as evidenced by the reported inhibitory effects on 6-OH-DA-induced nitrite and 8-iso-PGF_2α_ levels.

## 5. Conclusions

Concluding, the present study investigated the protective effects exerted by the water extracts of *G. glabra, U. rhyncophylla*, and *V. faba* in an experimental model of PD ex vivo. Our findings showed that all tested extracts were able to contrast the 6-OH-DA-induced increase in pro-oxidant biomarkers and DA turnover in the striatum, which could be related to their content in phenolic compounds. As a final remark, the present findings corroborate the rational for the traditional use of *V. faba*, *G. glabra*, and *U. rhyncophylla* extracts, as well as of their pharmacological association in order to improve their protective effects, including the increase in striatal steady levels of DA, which could be crucial in the management of the clinical symptoms of PD. In this regard, future in vivo studies prove necessary in order to verify the efficacy of these extracts.

## Figures and Tables

**Table 1:** HPLC–fluorimetric analysis of *G.* *glabra*, *U*. *rhyncophylla*, and *V*. *faba* water extracts. Data are reported as means ±S.D.

Phenolic Compound	*V. faba* (mg/g dry Extract)	*U. rhyncophylla* (mg/g Dry Extract)	*G. glabra* (mg/g Dry Extract)
Gallic acid	3.91 ± 0.35	30.80 ± 2.77	16.96 ± 0.68
Catechin	4.92 ± 0.59	6.11 ± 0.73	12.71 ± 0.99
Epicatechin	n.d.	n.d.	1.06 ± 0.05
Resveratrol	2.46 ± 0.25	25.25 ± 2.53	63.23 ± 7.14

n.d.: not determined.
